# Lipid raft/caveolae signaling is required for *Cryptococcus neoformans *invasion into human brain microvascular endothelial cells

**DOI:** 10.1186/1423-0127-19-19

**Published:** 2012-02-08

**Authors:** Min Long, Sheng-He Huang, Chun-Hua Wu, Gregory M Shackleford, Ambrose Jong

**Affiliations:** 1 Divisions Hematology-Oncology, The Saban Research Institute, Children's Hospital Los Angeles, Department of Pediatrics, Keck School of Medicine, University of Southern California, Los Angeles, CA 90027, USA; 2 Division of Infectious Diseases, The Saban Research Institute, Children's Hospital Los Angeles, Department of Pediatrics, Keck School of Medicine, University of Southern California, Los Angeles, CA 90027, USA; 3 Department of Microbiology, Southern Medical University, Guangzhou, China

**Keywords:** *Cryptococcus neoformans *, caveolin-1, CD44, brain microvascular endothelial cells, lipid raft

## Abstract

**Background:**

*Cryptococcus neoformans *has a predilection for central nervous system infection. *C. neoformans *traversal of the blood brain barrier, composed of human brain microvascular endothelial cells (HBMEC), is the crucial step in brain infection. However, the molecular mechanism of the interaction between *Cryptococcus neoformans *and HBMEC, relevant to its brain invasion, is still largely unknown.

**Methods:**

In this report, we explored several cellular and molecular events involving the membrane lipid rafts and caveolin-1 (Cav1) of HBMEC during *C. neoformans *infection. Immunofluorescence microscopy was used to examine the roles of Cav1. The knockdown of Cav1 by the siRNA treatment was performed. Phosphorylation of Cav1 relevant to its invasion functions was investigated.

**Results:**

We found that the host receptor CD44 colocalized with Cav1 on the plasma membrane, and knockdown of Cav1 significantly reduced the fungal ability to invade HBMEC. Although the CD44 molecules were still present, HBMEC membrane organization was distorted by Cav1 knockdown. Concomitantly, knockdown of Cav1 significantly reduced the fungal crossing of the HBMEC monolayer *in vitro*. Upon *C. neoformans *engagement, host Cav1 was phosphorylated in a CD44-dependent manner. This phosphorylation was diminished by filipin, a disrupter of lipid raft structure. Furthermore, the phosphorylated Cav1 at the lipid raft migrated inward to the perinuclear localization. Interestingly, the phospho-Cav1 formed a thread-like structure and colocalized with actin filaments but not with the microtubule network.

**Conclusion:**

These data support that *C. neoformans *internalization into HBMEC is a lipid raft/caveolae-dependent endocytic process where the actin cytoskeleton is involved, and the Cav1 plays an essential role in *C. neoformans *traversal of the blood-brain barrier.

## Background

*Cryptococcus neoformans *is commonly found in the environment, such as in the soil and in bird droppings. These fungal cells may be inhaled and deposited into the lungs and into the blood stream, providing a path for *C. neoformans *to reach the brain. In the case of the immunocompromised, this fungus could cause life-threatening cryptococcal meningitis [1,2]. In the world today, cryptococcal infection has become the most common fungal pathogen of the central nervous system [3]. In order to cause meningoencephalitis, the fungal cells must cross the blood-brain barrier (BBB). A special type of cell constitutes the BBB, the brain microvascular endothelial cells (BMEC), which are characterized by extremely tight intercellular junctions [4]. The large surface of BMEC exposed to blood circulation (~20 m^2 ^per human brain) underscores the importance of the BBB as the critical limiting factor for *C. neoformans *brain invasion.

Infection of *C. neoformans *into human brain microvascular endothelial cells (HBMEC) requires adherence to the host cell's surface in order to resist the flow of blood. It is generally accepted that the fungal capsule is the major virulence factor of this pathogen (reviewed by [5]). Real-time images have shown that the *C. neoformans *cells may become trapped within the microvessels in the brain, and subsequently, enter into the brain through the parenchyma [6]. Several mechanisms are possible for this transmigration process [6-12]. For example, hyaluronic acid (HA), produced by the hyaluronic acid synthase gene (*CPS1*) of *C. neoformans*, plays a role in the adhesion of the fungal cells to endothelial cells [9,13]. We have demonstrated that HBMEC CD44 is the primary receptor for *C. neoformans *infection [14]. Accordingly, the adherence of *C. neoformans *in the circulating blood to BMEC may be secured by the *C. neoformans*-produced HA and HBMEC CD44 interaction (the adhesion step). Then, the fungal cell triggers host signaling pathway(s) to facilitate its internalization (the invasion step). One feature of this process is the ability of C*. neoformans *to induce morphological changes in HBMEC, such as: membrane ruffling, irregular nuclear morphology and swelling of the mitochondria and the ER [15]. These findings suggest that *C. neoformans *is able to induce actin cytoskeletal reorganization of the host cells. Furthermore, activation of host PKCα is essential for *C. neoformans *internalization into HBMEC [10]. During *C. neoformans *infection, PKCα activation by phosphorylation is induced and PKC enzymatic activity is detected in the HBMEC membrane fraction. PKCα activation is a *CPS1*/CD44-dependent process. Blockage of PKCα function attenuates actin filament activity during *C. neoformans *invasion. Moreover, treatment with cytochalasin D, an actin disrupting reagent, can effectively block *C. neoformans *invasion into HBMEC *in vitro *[10]. Thus, the host PKCα action on *C. neoformans *internalization into HBMEC may be mediated via actin filament activity.

*C. neoformans *invasion into HBMEC is sensitive to the filipin treatment [14], which extracts cholesterol from membrane lipid rafts. It is generally accepted that endocytosis is mediated by membrane lipids or rafts. We have found that, in the presence of *C. neoformans*, the HBMEC lipid raft marker ganglioside GM1 can influx into the cytosol and accumulate in the perinuclear region [16]. The uptake of GM1 suggests the involvement of lipid raft endocytic pathways. An intriguing question raised is how *C. neoformans *builds its distinct uptake mechanism via lipid raft endocytic routes. This question is complicated by the fact that endocytosis is comprised of multiple mechanisms, i.e., caveolae-dependent, clathrin-dependent mechanisms, pinocytosis, macropinocytosis, and phagocytosis [17-19]. Upon *C. neoformans *and HBMEC engagement, a subpopulation of CD44, Cav1, and actin translocates to the host membrane rafts [14]. This link to dynamic caveolin trafficking suggests that caveolae may play a role during the adhesion and entry of *C. neoformans *at HBMEC membrane rafts.

Caveolae are small invaginations in the plasma membrane that mediate multiple functions, including signal transduction and endocytosis [19,20] and are characterized by the marker protein caveolins [21]. The mammalian caveolin family contains three members: caveolin-1, -2, and -3. Caveolin-2 is unable to form oligomers without Cav1. Caveolin-3 (M-caveolin) is a muscle specific member. Thus, Cav1 could be the critical caveolin in HBMEC. Cav1 is also known as VIP21 (Vesicular Integral-membrane Protein of 21 kD); indeed, the protein appears not only in the plasma membrane, but also on the Golgi-apparatus and vesicular membrane structures. The N-terminal region Cav1 contains the caveolin scaffolding domain (CSD; residues 82-101), which is essential for the formation of caveolin oligomers as well as interaction with other proteins. Abundant information reveals that caveolin participates in many important cellular processes, including vesicular trafficking, cholesterol homeostasis, cell adhesion and apoptosis [22]. Caveolins also interact with multiple signaling molecules, and so, it is believed that caveolins serve as scaffolding proteins for the integration of signal transduction. At least 37 proteins have been identified to interact with the CSD domain of caveolin [23]. Moreover, some reports suggest that the activity of caveolins seems to be dependent on their specific post-translational modifications, which then allow them to modulate cellular signaling cascades. For example, the palmitoylation of Cav1 at a single site (Cys156) was shown to be necessary for the coupling of Cav1 to c-Src tyrosine kinase [24]. Also, phosphorylation at Tyr14 is essential for caveolin association with SH2 or PTB domain-containing adaptor proteins, such as GRB7 [25]. It is suggested that Cav1 stabilizes caveolae at the plasma membrane and thereby acts as a negative regulator of the internalization of caveolae or lipid raft-like membrane domains [25]. Upon phosphorylation of caveolin, caveolae are induced and then internalized by endocytosis. However, the detailed mechanism of this process remains to be further elucidated.

Our previous studies demonstrated that *C. neoformans *adhesion to HBMEC takes place on the lipid rafts and Cav1 may take part in the invasion process [14]. However, the role of caveolin during *C. neoformans *infection has never been tested. In this report, we first examined the impact of Cav1-knockdown on host receptor CD44 functions and on lipid rafts. Concurrently, the knockdown of Cav1 significantly reduced *C. neoformans *invasion and the crossing of the HBMEC monolayer *in vitro*. We further examined the phosphorylation regulation of Cav1 in response to *C. neoformans *invasion. Our studies are the first to demonstrate that phosphorylated Cav1 migrates inward in HBMEC along with actin filaments but not with the microtubule cytoskeleton. Together, our results suggest that *C. neoformans *invasion into HBMEC is linked to the lipid raft/caveolae-dependent endocytotic process and that Cav1 plays an essential role during this invasion process.

## Methods

### *C. neoformans *strains, media and cultures

**Figure 1:**
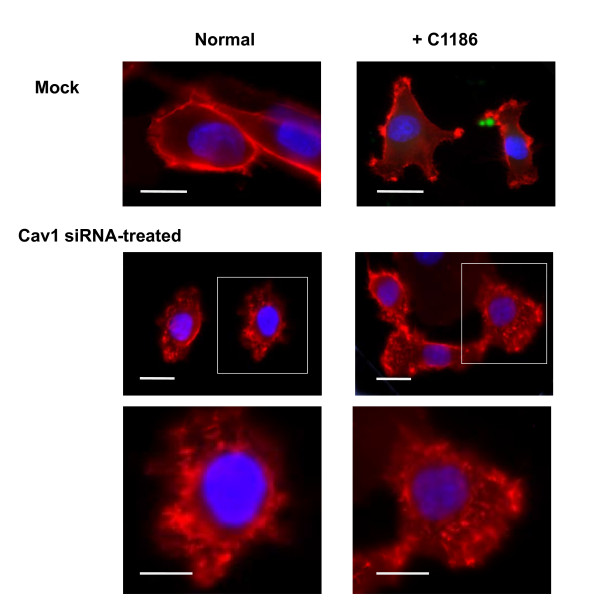
**Effect of CD44 membrane distribution on the Cav1 knockdown HBMEC**. DAPI was used to stain nuclear DNA (blue) and CD44 was stained with the anti-CD44 monoclonal antibody and rhodamine conjugated secondary antibody (red). **Top panel**: membrane distributions of CD44 without (left panel) or with (right panel) the incubation of green *C. neoformans *strain C1186. **Lower panel**: HBMEC were treated with Cav1 siRNA in the presence (right column) or absence (left column) of *C. neoformans *cells (strain C1186). Magnified images were boxed and shown in the bottom panel. Bar: 20 μm.

**Figure 2:**
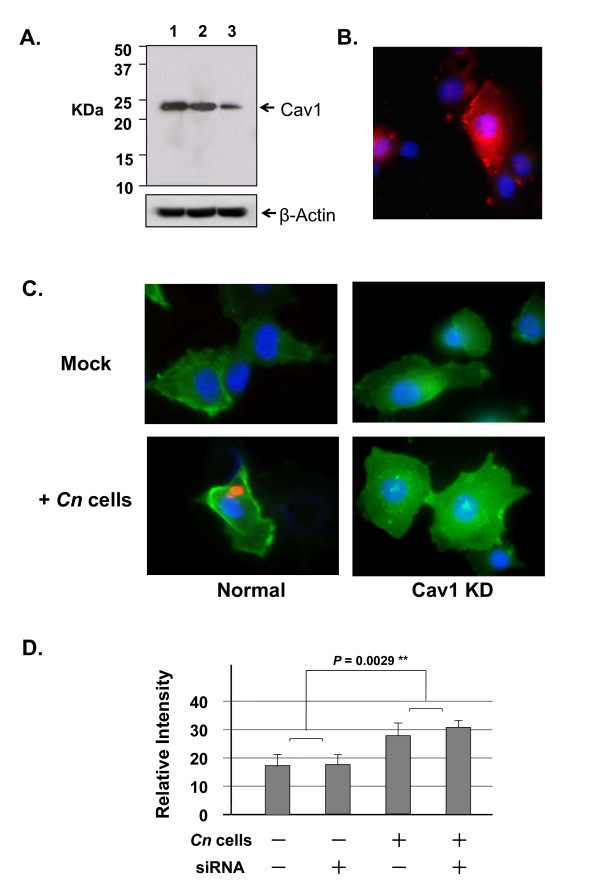
**Effect of Cav1 siRNA on HBMEC**. (A) A Western blot shows the Cav1 proteins from HBMEC with the following treatments: lane 1: mock; lane 2: random oligonucleotides; and lane 3: Cav1 siRNA. (B) An immunofluorescence microscopic image shows the reduction of Cav1 in the siRNA-treated HBMEC. Most cells show only DAPI stain, and one HBMEC still has some Cav1 signals (red). (C) The membrane raft marker ganglioside GM1 of HBMEC was stained with CTxB-FITC (green) and a *C. neoformans *cell (strain B-4500FO2) was stained with anti-GXM monoclonal antibody and rhodamine conjugate (red) (lower panel). The upper panels (Mock) are the controls without incubation with *C. neoformans *cells (strain B-4500FO2) (+*Cn *cells). The right column is HBMEC with Cav1 siRNA treatment (Cav1-KD) vs. the control (left column). (D) The Metamorph program associated with our fluorescence microscope was used to scan images with 5~10 random fields, and then used the statistical package GradPad Prime 5 to quantitate the readings. Analysis of variance shows significant increases in the GM1 signals in the presence of *C. neoformans *cells (*P *= 0.0029), but no different in the siRNA treated samples.

The *C. neoformans *strain B-4500FO2 is the parental strain used mainly in this report [9,13]. C559 is an isogenic deletion mutant of the *CPS1 *gene, which is derived from strain B-4500FO2. C1186 is a strain B-4500FO2 with a stable expression of GFP. This strain was used in Figure 1 for the purpose of showing the interaction between *C. neoformans *(green) and HBMEC (red). In Figure 2C, the Cav1-GFP showed a green signal, therefore strain B-4500FO2 was used to stain with anti-GXM antibody and the 2^nd ^antibody conjugated with rhodamine (red). *C. neoformans *cells were grown aerobically at 30°C in 1% yeast extract, 2% peptone and 2% dextrose (YPD broth)(Difco Laboratories, Detroit, MI). Cells were harvested at early log phase, washed with phosphate-buffered saline (PBS) and resuspended in Hams-F12/M199 (1:1, v:v), 5% heat-inactivated fetal bovine serum (experimental medium), and 1% human serum. The *Cryptococcus *cell number was determined by direct counting from a hemocytometer [26].

### HBMEC culture

HBMEC were isolated and cultured as described previously [10]. HBMEC cultures were maintained in RPMI 1640 medium containing 10% heat-inactivated fetal bovine serum, 10% NuSerum (BD Bioscience, Bedford, MA), 2 mM glutamine, 1 mM sodium pyruvate, 100 μg/ml streptomycin, 100 units/ml penicillin, essential amino acids, and vitamins. The cells were washed before performing experiments. The expression vector with the Cav1-GFP was obtained from Addgene, Inc (plasmid #14433). The plasmid was transfected into HBMEC using neomycin for selection.

### Immunofluorescence microscopy

Samples for immunofluorescence microscopy were prepared as follows. HBMEC were plated onto glass coverslips (22 mm, square), which had been previously coated with type I collagen from rat tail (Upstate, 5-10 μg/cm^2^) in an 8-well square culture system (Nalgene Nunc). HBMEC (~5 × 10^4 ^cells) were seeded onto one coverslip 24 h prior to the experiment. HBMEC were prewashed four times with PBS, then fixed with 2% formaldehyde/PBS (v:v) for 30 min at room temperature. After additional three-washes with PBS, the HBMEC were blocked with 5% milk/PBS for 30 min and then incubated with antibody or proper reagent at 4°C overnight. Anti-CD44 monoclonal antibody was purchased from Santa Cruz (used at 1:500 dilution); Cholera toxin subunit B (CTxB)-FITC was purchased from Sigma Chem. Co. (#C1655, 1~2 μg/mL); anti-Cav1(tyr-14) was purchased from Cell Signaling Technology (#3251); actin filaments were stained with phalloidin-rhodamine conjugate (Sigma Chem Co. cat #P1951), and anti-tubulin antibody was obtained from Sigma Chem Co., (#T5168). Anti-GXM monoclonal antibody 18B7 was used to stain the fungal cell (kindly provided by Dr. Casadevall, Albert Einstein College of Medicine). The coverslips were then washed 4 times with PBS, then 1% BSA/PBS and/or anti-mouse IgG FITC conjugate (1:100 dilution) was added into each well for 1 h at 4°C. Another three washes were applied, then a drop of Vectashield mounting solution containing DAPI (Vector Laboratories; H-120) was used to seal the coverslips onto slides. Samples were examined under a fluorescence microscope at the Congressman Dixon Cellular Imaging Core Facility, Children's Hospital Los Angeles.

### *In vitro *adhesion and invasion assays

**Figure 3:**
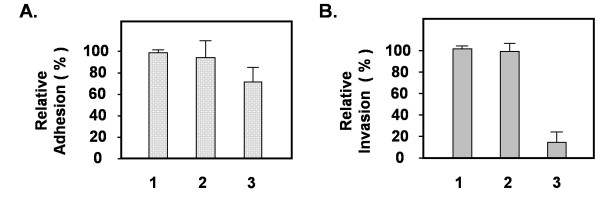
**Effect of *C. neoformans *on the adhesion and invasion of HBMEC in the Cav1 knockdown HBMEC**. HBMEC cells were pretreated with (**1**) mock, (**2**) random oligo control, and (**3**) Cav-1 siRNA, before (A) the adhesion and (B) the invasion analyses. The treated samples were incubated *C. neoformans *strain C1186 (10^5 ^cells) for 3 hr and then the slides were prepared for immunofluorescence imaging (see Materials and Methods). The mock sample (lane **1**) was designated as 100%, and the effects of siRNA were indicated by percentage over the control, respectively. Each bar represents the average of four different experiments ± SD with P < 0.01. Detailed adhesion and invasion assays are described in Materials and Methods.

Immunofluorescence microscopy was used for the *in vitro *adhesion and invasion assays, as described previously [10]. Briefly, the HBMEC were probed with β-actin using phalloidin-rhodamine conjugate to display a red background. *C. neoformans *C1186 cells show a bright green fluorescence. After 3 hr incubation, the slides were prepared for immunofluorescence imaging. Individual green *C. neoformans *cells were observed clearly by immunofluorescence microscopy. For those adherent *C. neoformans *cells, the green signals from C1186 partially overlapped with the red-stained HBMEC and thus displayed the green/yellow signals. For invaded *C. neoformans *cells, the internalized *C. neoformans *cells bearing GFP were totally overlapped with red background and thus showed a complete yellow signal. The total adherent (Figure 3A) or invaded (Figure 3B) *C. neoformans *cells of the untreated samples were designated as 100%. Five random regions in the chamber slide in each sample were counted under the microscope. The assay for each experiment was reproduced at least 3 times.

### *In vitro *transcytosis assay

*C. neoformans in vitro *transcytosis assays were performed as described previously [14,15]. Briefly, HBMEC were cultured on collagen-coated Transwell polycarbonate tissue culture inserts with a pore diameter of 12 μm (Corning Costar) for 24 h. Triple samples of HBMEC were pretreated with Cav1 siRNA (0, 10 and 20 pmoles in 0.5 mL culture medium) individually for an additional 48 h. HBMEC were polarized and exhibited a trans-endothelial electrical resistance (TEER) of 250~300 μΩ per cm^2^, as measured with an Endohm volt/ohm meter (World Precision Instruments). On the morning of the assay, HBMEC monolayers were washed with experimental medium and 10^6 ^*Cryptococcus *cells were added to the upper chamber (total volume 500 μl); they were then incubated at 37°C. At 4 and 8 h, samples (100 μl) were taken from the lower chamber and plated for counting of CFU. The lower chamber was replenished with 100 μl fresh culture medium. Simultaneously, the integrity of the HBMEC monolayer was assessed by measurement of the TEER. Three measurements were made at each time-point for each sample.

### Cav1 knockdown experiments

**Figure 4:**
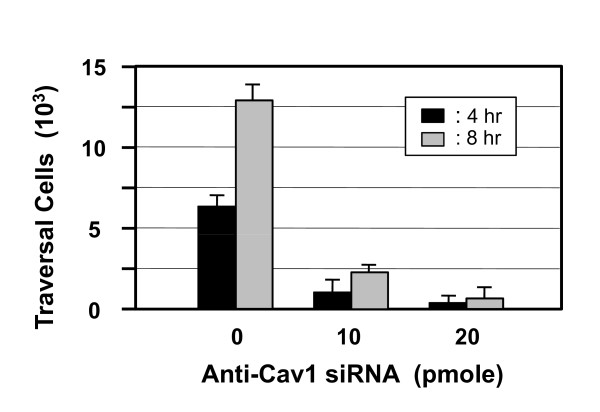
**Effect of Cav1-knockdown HBMEC on *C. neoformans *transmigration across the monolayer**. HBMEC (10^4 ^cells) were seeded on the collagen-coated transwells for four days, until transendothelial electric resistance (TEER) reached > 250 μΩ/cm^2^. The coated cultures were treated with control oligo or Cav1 siRNA, either 10 or 20 pmoles, in the 0.5 mL upper chamber, for 2 days before the transcytosis studies. CFU were counted from the lower chamber of the transwell at 4 and 8 h after the addition of *C. neoformans *(n = 3). Analysis of variance shows significant decreases in transcytosis ability in the Cav1 knockdown HBMEC (*P *= 0.011).

Cav1 siRNA was purchased from Santa Cruz Biotech (#sc-29241), and a control oligo (#sc-36869) was used in parallel as the control. Cav1 siRNA treatment is according to the manufacturer's protocol. Briefly, 0, 10, 20 pmoles siRNA (or indicated concentrations) was transfected into HBMEC, using SuperFection TM siRNA Transfection Reagent (cat.# SL100559) from SignaGen^® ^Lab (Gaithsburg, MD). After 5 h, the culture was replaced with fresh medium and grown for another 24 h. Then the HBMEC were washed with PBS three times before the experiments. One set of sample was used to detect the Cav1 protein level (Figure 2A), and another set of treated HBMEC was used for several studies. Similar preparations were performed for immunofluorescence microscopic studies (Figures 1, 2B, &2C), *in vitro *adhesion and invasion assays (Figure 3), and the transcytosis assay (Figure 4).

### Statistical analysis

The statistical analysis of the data from our *in vitro *studies involved analysis of variance (ANOVA). The dependent variable was the associated percent of cells or CFU while the independent fixed factors were the treatments (Cav1 siRNA). Raw data was entered into EXCEL files and automatically converted to statistical packages. ANOVA and co-variates were followed by the Newmann-Keuls test, to determine the statistical significance between the control and treatment groups. *P *< 0.05 was considered to be significant.

## Results

### Colocalization of Cav1-GFP and CD44 on the surface of HBMEC

**Figure 5:**
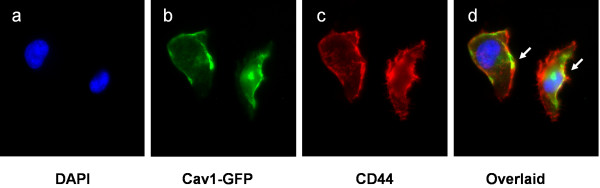
**Colocalization of Cav1-GFP and CD44 on the surface of HBMEC**. HBMEC were transformed with Cav1-GFP (1b, green). DAPI was used to stain nuclear DNA (1a, blue), and CD44 was stained with an anti-CD44 monoclonal antibody and Rhodamine conjugate (1c, red). An overlaid image was displayed on the right, showing some yellow signals (1d, arrows).

We have previously demonstrated that the HBMEC CD44 plays the key role as the host receptor during *C. neoformans *invasion [14]. Upon *C. neoformans *association, both CD44 and Cav1 translocate to the surface membrane rafts. However, the relationship between CD44 and Cav1 is still not clear. As Cav1 is a major structural and functional protein component of caveolae/lipid rafts, we would like to clarify the relationship of these two proteins during *C. neoformans *infection. We first detected whether there is a colocalization between Cav1 and CD44 molecules on the membrane of HBMEC. DAPI stain (blue) was used to locate HBMEC cells (Figure 5a). Cav1-GFP has been verified as a reliable marker for endogenous Cav1 [21], thus we used it to represent its localization (Figure 5b). In general, Cav1 can be found on the plasma membrane, peri-centrosomal caversomes around the nucleus, and caveolae vesicles in the cytosol. Its localization in HBMEC is similar to the observation in CHO cells [21]. CD44 is located both on the plasma membrane and in the cytosol (Figure 5c). In an overlaid image, there are colocalizations of Cav-1-GFP and CD44 in some areas on the plasma membrane (10~15%) (Figure 5d, arrows), as illustrated by the bright yellow signals. No or little colocalization of CD44 and Cav1 was observed inside the HBMEC.

### Effect of anti-Cav1 siRNA on HBMEC during *C. neoformans *infection

Infection of *C. neoformans *into HBMEC first requires initial adherence to the host cell surface resulting in the triggering of the host cell's signaling pathway(s) to facilitate its internalization. We investigated whether the knockdown of Cav1 with siRNA would affect CD44 function and/or *C. neoformans *invasion into HBMEC. A random oligo and buffer alone were used in parallel as controls. After 24 h of treatment, one set of samples was used to detect the Cav1 protein level (Figure 2A). Under our experimental conditions, ~60% of the endogenous Cav1 was knocked down in the Cav1 siRNA-treated HBMEC (Figure 2A, lane 3) compared to the controls (Figure 2A, lanes 1 & 2). Another set of samples was used for immunofluorescence images to examine Cav1 using anti-Cav1 antibodies with rhodamine conjugate (red) in the Cav1-knockdown HBMEC. As shown in Figure 2B, there are several DAPI stains void of Cav1 signals; presumably these are Cav1-knockdown HBMEC cells. Although limited, Cav1 signal can be detected in some HBMEC cells. These signals are either significantly reduced or display a more diffused distribution in HBMEC. The overall reduction of Cav1 signal is consistent with protein blotting studies (Figure 2A).

To further examine the alterations in Cav1-knockdown HBMEC, we used cholera toxin subunit B (CTxB)-FITC conjugate to locate the membrane raft marker ganglioside GM1. In the absence of *C. neoformans*, the GM1 signal was weak (Figure 2C, left, upper panel). Correspondingly, the signal increases, particularly on the membrane portion, in the presence of *C. neoformans*. However, in the Cav1 siRNA-treated HBMEC, the signal of GM1 was weak and void of membrane staining (Figure 2C, right panels). Despite the signal increases in the presence of *C. neoformans *cells, there is no noticeable redistribution of GM1 on the surface of Cav1 KO HBMEC in the siRNA treated HBMEC (Figure 2C, right panels). Analysis of variance shows significant increases in GM1 signal in the presence of *C. neoformans *cells (*P *= 0.0029), but not much different in the siRNA treated samples (Figure 2D). Thus, lack of Cav1 might prevent the reorganization of membrane lipid rafts of HBMEC in response to *C. neoformans *infection.

### Perturbation of CD44 distribution on HBMEC plasma membrane after Cav1 siRNA treatment

We next explored if there was any effect on the distribution of CD44 on the Cav1 knockdown HBMEC. Through the immunofluorescent staining, the control (CD44 without *C. neoformans *cells) is depicted by a vivid contour on the membrane (Figure 1, upper, left panel). In contrast, the CD44 signals increase and clusters on the membrane in the presence of *C. neoformans *strain C1186 cells (Figure 1, upper, right panel), particularly around the *C. neoformans *adhesion site. Interestingly, the Cav1 knockdown HBMEC displayed a very unique phenotype *i.e*., the CD44 stains become irregularly diffused throughout the surface (Figure 1, bottom panels), regardless of the presence or absence of *C. neoformans *cells (Figure 1, left vs. right bottom panels). The magnified images were displayed in the lower bottom panels. It seems that CD44 loses its organization and becomes concentrated in islands on the surface of HBMEC after Cav1 is knocked down. However, the total CD44 intensity seems unchanged. This unique phenotype leads us to inquire: (1) can these CD44 molecules in the Cav1 siRNA-treated HBMEC still act as functional receptors for the adherence of *C. neoformans *cells, and (2) can *C. neoformans *cells still invade into Cav1 siRNA-treated HBMEC? These questions are addressed below.

### Effect of Cav1 siRNA on the adhesion and invasion of *C. neoformans *into HBMEC

We speculated that knockdown of Cav1 may have some profound effects on the CD44 receptor function, therefore, we used *in vitro *adhesion and invasion assays to study *C. neoformans *infection on Cav1 knockdown HBMEC [14]. Untreated HBMEC or HBMEC treated with control oligo were used in parallel (Figure 3A, lanes 1 and 2). We found that the association of *C. neoformans *with HBMEC was reduced only slightly in the Cav1 siRNA-treated HBMEC (Figure 3A, lane3). Presumably, *C. neoformans *cells can adhere to the surface of HBMEC, as the CD44 molecules are still present. On the other hand, the number of invaded fungal cells was significantly reduced in Cav-1 siRNA-treated HBMEC (Figure 3B, lane 3), suggesting that *C. neoformans *cells attached on the surface of HBMEC but failed to internalize into the Cav1 knockdown host cells. In this regard, the interaction between fungal cells and the host CD44 appears to be effective; however, the Cav1 siRNA-disrupted of caveolae/lipid raft structures may not be able to organize into an effective fungal entry site. Alternatively, the reduction of Cav1 molecules may not be able to trigger sufficient host signaling for *C. neoformans *internalization.

### Effect of Cav1 siRNA on *C. neoformans *traversal across the HBMEC monolayer *in vitro*

We have set up an *in vitro *blood-brain barrier model to study how *C. neoformans *cells traverse the HBMEC monolayer [10,14]. HBMEC were seeded on the collagen-coated transwell filter and treated with the control oligonucleotide and Cav1 siRNA in parallel for comparison. The transendothelial electric resistance (TEER) was used to monitor the intactness of the tight junction of HBMEC monolayer. In general, the TEER was maintained around 250~300 μΩ throughout the experiments, indicating that the tight junctions were maintained during the study (data not shown). Our results showed the ability of *C. neoformans *to traverse the monolayer was significantly decreased in the absence of Cav1 (Figure 4), suggesting that Cav1 is crucial for the *C. neoformans *penetration through the blood-brain barrier. Taken together, our results support that Cav1 is not only required for the integrity of membrane raft structure (Figure 1), but also responsible for *C. neoformans *internalization (Figures 3). These functions are crucial for *C. neoformans *cells to cross the blood-brain barrier (Figure 4), a process which may subsequently lead to central nervous system infection.

### Distribution of phosphorylated Cav1 on HBMEC during *C. neoformans *infection

**Figure 6:**
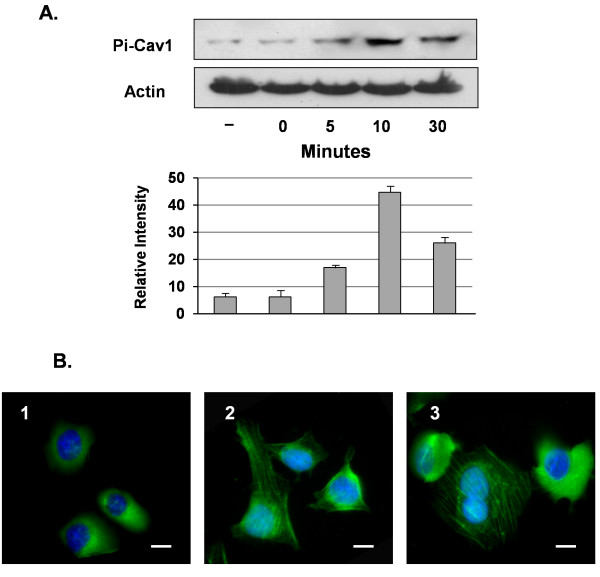
**Induction of Cav1 phosphorylation during *C. neoformans *infection**. (A) HBMEC were infected with *C. neoformans *B-4500FO2 for different time periods, and then cell lysates (50 μg) were blotted and probed with an anti-phospho-Cav1 (Tyr-14) antibody. A time course study of Cav1 phosphorylation at 0, 5, 10, and 30 min is shown. β-actin was used as the loading control (second row). The ECL films (protein blots) were scanned by the software ImageMaster 2D Platinum 6.0 for quantitative and graphic analyses (lower panel). The total intensity of all bands is 100%. The relative intensity of the bands is indicated on the *y*-axis (n = 3). (B) Distribution of Pi-Cav1 during *C. neoformans *infection was examined by immunofluorescence microscopy. Untreated HBMEC were used as the control (1), or the HBMEC monolayer was incubated with 10^6 ^*C. neoformans *strain B-4500FO2 for (2) 5 min and (3) 30 min. Pi-Cav1 was stained with anti-phospho-Cav1 (Tyr-14) and second antibody-FITC conjugate (green). Nuclei were stained with DAPI (blue). Bar: 15 μm.

It has been suggested that, upon external stimulus, caveolin may be activated through its phosphorylation regulation [27,28]. Limited information is available regarding the Cav1 phosphorylation regulation in relation to pathogen invasions [29]. So, we explored this regulation in HBMEC during *C. neoformans *infection. We first performed the time-course studies of Cav1 during *C. neoformans *infection to evaluate its phosphorylation in HBMEC. Using anti-phospho-Tyr-14 Cav1 specific antibodies, we detected a basal level of phosphorylated Cav1 (Pi-Cav1) at the zero time point, suggesting that the internalization of caveolae is already active for certain normal cellular functions in HBMEC (Figure 6A). Upon *C. neoformans *treatment, the Pi-Cav1 increased at 5 min and peaked at least 5-fold above basal level at 10 min. Thus, *C. neoformans *is able to significantly induce Cav1 phosphorylation, presumably resulting in its activation.

We further used immunofluorescence microscope to investigate the Pi-Cav1 dynamics in response to *C. neoformans *infection. In the absence of *C. neoformans*, the basal level of the Pi-Cav1 could be observed in the perinuclear regions in the cytosol (Figure 6B-1). Unlike total Cav1 distribution shown in Figure 5, little or no Pi-Cav1 signal was detected in the plasma membrane of HBMEC. In the presence of *C. neoformans *(5 min), some membrane signals can be detected, but more significantly, many linear array structures of Pi-Cav1 signals were observed inside the HBMEC (Figure 6B-2). In a prolonged incubation (60 min), a mixed population of Pi-Cav1 staining can be observed (Figure 6B-3). In some cells, the cytosolic Pi-Cav1 displayed small vesicle structures (dots); however, the linear arrays are more obvious. In other cells, the perinuclear localization can be observed, suggesting the terminal location of Pi-Cav1. Thus, membrane Cav1 may be phosphorylated, internalized and accumulated in the perinuclear region of HBMEC. The linear structure may represent its functions in shuttling caveolae or caveolae-like membrane vesicles between plasma membrane and caveosome around the peri-centrosomal region [30].

**Figure 7:**
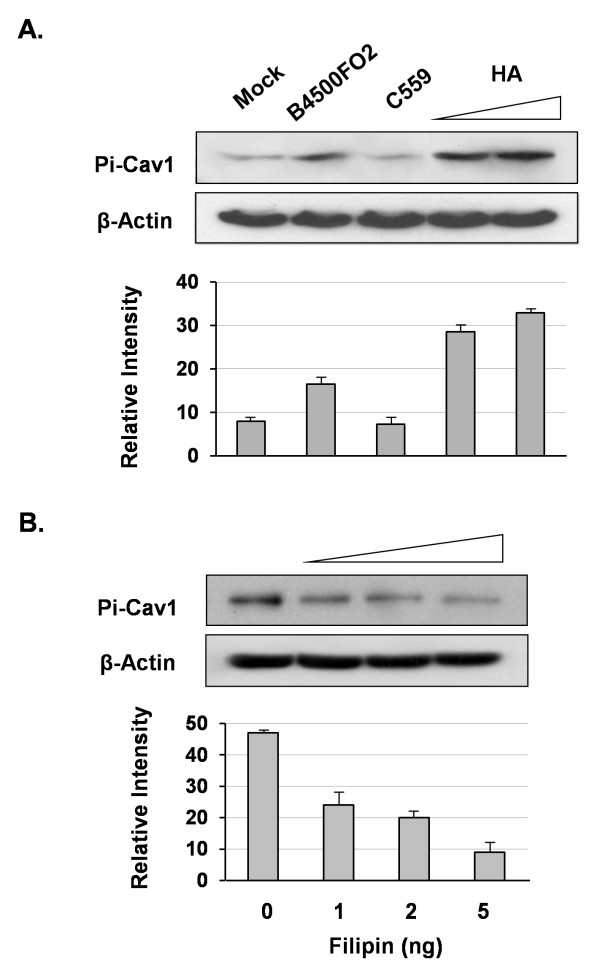
**Induction of Cav1 phosphorylation mediated by the HA-CD44 pathway**. (A) HBMEC were treated with 10^6 ^*C. neoformans *strains B-4500FO2 (*CPS1*^+^) or C559 (*cps1*Δ), or treated with 1 and 2 μg hyaluronic acid (HA) for 10 minutes (A). The cell lysates were prepared and immunoblotted for the detection of the phosphorylated form of Cav1 (top row). β-actin was used as the loading control (second row). The quantitative and graphic analyses (lower panel) were described in the legend of Figure 6. (n = 4) (B) HBMEC cells were pre-treated with filipin (0 to 5 ng) for 10 minutes prior to the treatment of 10^6 ^*C. neoformans *strain B-4500FO2 cells. The signals of Pi-Cav1 were then examined as described above.

To further examine its phosphorylation regulation, *C. neoformans *isogenic *CPS1 *strains were used. *C. neoformans *strain B-4500FO2 (*CPS1*^+^) could, but strain C559 (*cps1*Δ) could not, enhance the Pi-Cav1 signal (Figure 7A). Since C559 is defective in the biosynthesis of hyaluronic acid (HA), we then tested whether HA by itself could stimulate the phosphorylation of Cav1. As shown in Figure 7A (lines 4, 5), the Cav1 phosphorylation was increased in a hyaluronic acid-dependent manner. Thus, phosphorylation regulation of Cav1 is an HA-CD44 dependent process.

Filipin was used to perturb the membrane lipid raft structure on HBMEC. Cav1 phosphorylation was stimulated by *C. neoformans *B-4500FO2 first, but this induced phosphorylation level was decreased as the filipin concentration increased (Figure 7B). Therefore, the filipin treatment reduced an already induced level of phophorylation, and/or prevented the *C. neoformans*-induction of phosphorylation. These results suggest that Cav1 phosphorylation might take place at the membrane lipid rafts and/or that Cav1 phosphorylation requires an intact membrane raft structure.

### Colocalization of Pi-Cav1 with β-actin, but not with tubulin, during internalization

**Figure 8:**
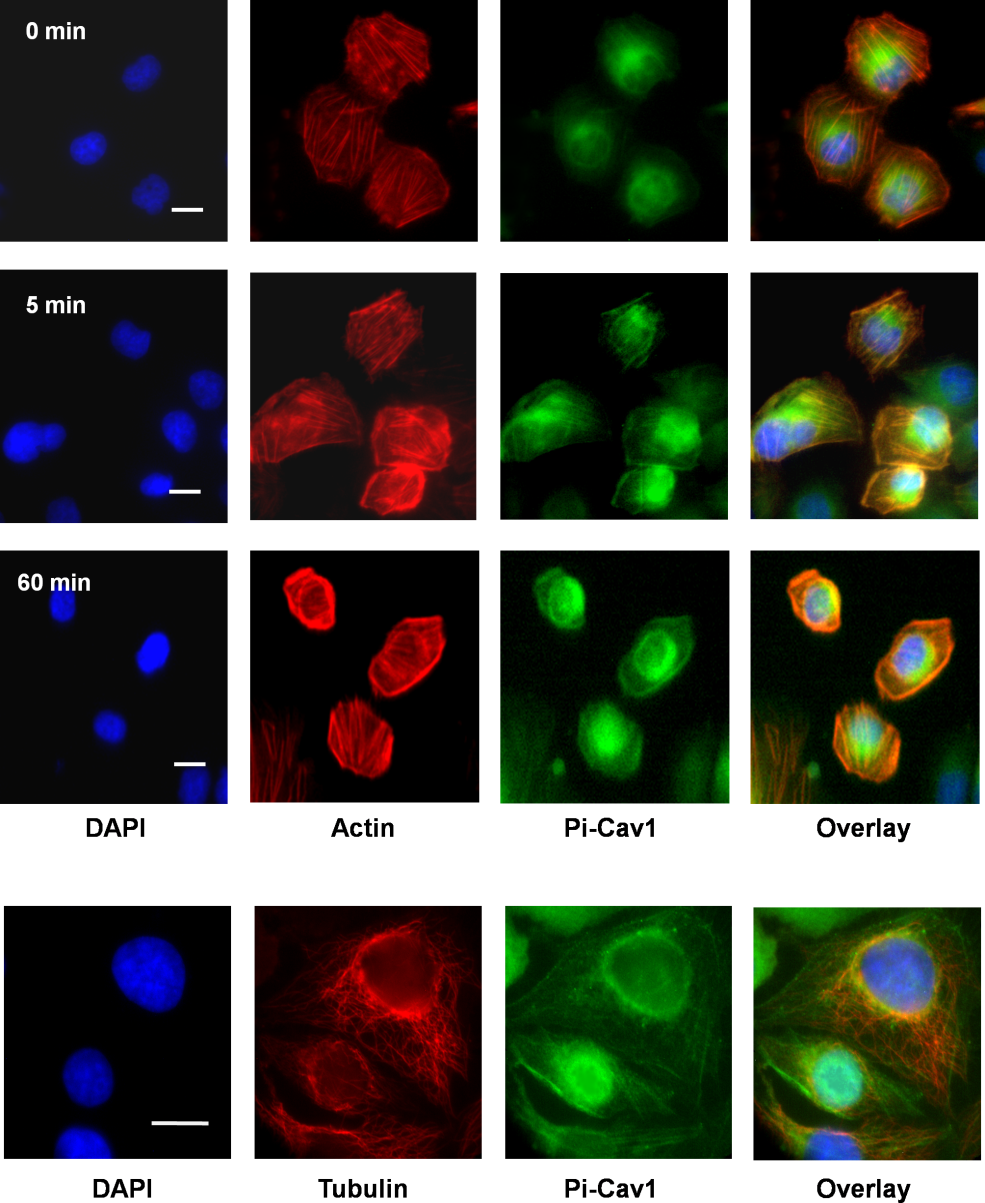
**Immunofluorescence microscopy to examine phospho-Cav1 and β-actin localization during *C. neoformans *infection**. The HBMEC monolayers were incubated with 10^6 ^*C. neoformans *strain B-4500FO2 for 0 min, 5 min and 60 min. In parallel, untreated HBMEC were used as a negative control, indicated as 0 min in the figure. β-Actin was stained with phalloidin-rhodamine conjugate (red) (second column) and the phosphor-Cav1 was stained with anti-phospho-Cav1 (Tyr-14) antibody and second antibody-FITC conjugate (green). Nuclei were stained with DAPI (blue). Bar: 15 μm. A similar study was performed in the bottom panel, in which tubulin was stained with anti-tubulin antibodies and rhodamine conjugate (second column) at the 60 min incubation time point.

During endocytosis, endosomal trafficking is required both the actin cytoskeleton and microtubules [21]. However, how they are linked to Pi-Cav1 dynamics has never been explored. To further explore the nature of the linear array structure observed in Figure 6B, we examined the relationship between Pi-Cav1, actin and the microtubules. HBMEC were incubated with B-4500FO2 (*CPS1*^+^) at 0, 5 and 60 min and then probed for nuclear DNA, β-actin and Pi-Cav1. DAPI stains nuclear DNA (blue) to locate the individual HBMEC cell (Figure 8, first column). β-Actin molecules were stained by the phalloidin-rhodamine conjugate (red) (Figure 8, second column), and Pi-Cav1 was stained with a specific antibody conjugated with FITC (green) (Figure 8, third column). At the 5 min incubation period, the effect of *C. neoformans *on β-actin (red) and phospho-Cav1 (green) could be observed. Both signals increased and thread-like structures became more obvious throughout the HBMEC. The stress fibers of actin filaments are well known, but the thread-like fibers of Pi-Cav1 have never been described. At 60 min, similar structures could be observed, except that β-actin showed stronger signals on the plasma membrane, while Pi-Cav1 signals localized more in the perinuclear region, presumably in caversomes around the pericentrosomal region. In all cases, a colocalization of β-actin and Pi-cav1 on the plasma membrane and pericentrosomal region close to the nucleus, as thread-like structures, can be observed (Figure 8, 4th column). This can be vividly observed as the yellow/orange signals in the overlaid images. This result raised an interesting possibility that the trafficking of Pi-Cav1 may be linked to the β-actin cytoskeleton system.

A similar study was performed to study the relationship between tubulin and Pi-Cav1. Although tubulin staining displayed a random, thread-like network throughout the cytosol in HBMEC, there was no overlapping with Pi-Cav1's long thread-like structures (Figure 8, bottom panels). In the perinuclear staining, there might be some overlap between Pi-Cav1 and tubulin, but it was not significant. Thus, Pi-Cav1 trafficking inside the HBMEC may be a microtubule-independent process.

## Discussion

*C. neoformans *needs to cross the BBB in order to infect the central nervous system. Unraveling the biological mechanism of *C. neoformans *invasion across the BBB is crucial to understanding the microbial pathogenesis of this disease. Previous reports have suggested that there are several possible pathways for *C. neoformans *to enter the host brain [7-9,11,12,14]. If multiple mechanisms exist, then the important question is: which is the major route of cerebral invasion by *C. neoformans*? Several *in vivo *studies have provided clues to this mystery. For example, *C. neoformans *cells could be found in mouse brains after only 5 min following intravenous injection with a low dose of pathogen (10^4 ^H99 cells per mouse) [31]. But extravasation of horseradish peroxidase through the BBB could only be detected in mouse brains 6 h after injection with a high dose of pathogen (10^7 ^H99 cells per mouse) [7]. Based on the above information, damage to the tight junction or other paracellular mechanism may not be the major method that *C. neoformans *uses to cross the BBB. *In vitro*, transmission electron microscopic images show that *C. neoformans *triggers the formation of microvillus-like membrane protrusions in HBMEC within a short time (15 to 30 min), after which *C. neoformans *cells were internalized by the cells. They penetrated across the HBMEC monolayers via a transcellular pathway without affecting monolayer integrity [31]. One possible mechanism for this is that fungal transcytosis may be mediated by the fungal hyaluronic acid and host HBMEC CD44 receptor [13,14]. Collectively, there is an active invasion process(es) allowing fungal cells to effectively traverse the intact BBB. To explore the mechanism of transcellular migration during cerebral invasion by *C. neoformans*, we studied the host response during this process. In this report, we investigated the interplay among fungal cells, host CD44, membrane lipid rafts and Cav1 to analyze the mechanism of adhesion and invasion by this fungus.

CD44 is the *C. neoformans *receptor on the lipid rafts/caveolae of HBMEC [14]. Here we initially demonstrated that CD44 was colocalized with Cav1 on the surface of HBMEC (Figure 5), supporting the notion that CD44 exerts its functions on lipid rafts. Lipid rafts are enriched in cholesterol, sphingolipids and raft proteins. These highly dynamic membrane structures may have a potential role as signaling platforms by clustering receptors used by pathogens. Caveolae are a special type of membrane lipid rafts. We therefore investigated whether their major component, Cav1, is involved in the *C. neoformans *invasion process. We found that the lipid raft marker ganglioside GM1 (Figure 2) can cluster around the *C. neoformans *adhesion site. Knockdown of Cav1 perturbed GM1 distribution (Figure 2), and, more interestingly, the CD44 proteins became randomly distributed and clustered into irregular islands on the surface of HBMEC (Figure 1B). This unique morphology has never been described in any cell type. As such, *C. neoformans *invasion into HBMEC is impaired (Figure 3B). The results suggest that Cav1 may play an important structural role in the reorganization of CD44, optimizing it to function as a receptor during *C. neoformans *invasion. Furthermore, transcytosis of *C. neoformans *was significantly reduced in the Cav1 knockdown HBMEC (Figure 4). Together, CD44 might be anchored on the lipid rafts/caveolae to form an entry site on the surface of HBMEC during *C. neoformans *infection. At present, there is no evidence to show a direct interaction between CD44 and Cav1. On the other hand, it has been documented that there is a direct interaction between PKCα and Cav1 [32], and that activation of PKCα on the plasma membrane is required for *C. neoformans *invasion into HBMEC [10]. The relationship between Cav1 and PKCα for *C. neoformans *invasion is interesting, but still needs to be further elucidated.

One interesting question regarding *C. neoformans *invasion is that the *C. neoformans *cell is able to hijack the endocytic signaling in HBMEC in order to facilitate its internalization [16]. Notice that endocytosis comprises multiple mechanisms, i.e., caveolae-dependent, clathrin-dependent, pinocytosis, macropinocytosis, and phagocytosis [17-20,30]. In this report, we first demonstrated the involvement of Cav1 in *C. neoformans *infection. Perturbation of Cav1 function by knockdown of Cav1 (Figures 3 and 4) can substantially reduce the ability of fungi to invade HBMEC. Cav1 is the major protein constituent of caveolae [23]. Thus, our results support the notion that *C. neoformans *may utilize the lipid raft/caveolae endocytic pathway for its invasion. Caveolae are small invaginations in the plasma membrane that mediate multiple functions, including signal transduction and endocytosis [19,30]. Increasing evidence shows that pathogens are able to use lipid rafts or caveolae as cell surface platforms for interacting with, binding to and possibly entering into the host cells [33]. Typical caveolae are 70 to 100 nm in diameter. Sphingolipid binding toxins (cholera toxin CTxB and shiga toxin) and viruses (including SV40) can be carried inside the caveolae cargo, whereas bacteria (> 1 μm in diameter) are much larger than caveolae, which might thus not be suitable to accommodate their engulfment. In this case, the caveolae do not provide the entry route *per se *[34], but offer signaling platforms required for the bacterial invasion. The size of *C. neoformans *is ~5 μm or larger in diameter, depending on its capsule size. It is conceivable that entry of this organism is mediated by its association with lipid rafts, rather than by its enclosure within caveolae *per se*, and that host Cav1 plays a crucial role of *C. neoformans *invasion into HBMEC.

We further studied how Cav1 was involved in this invasion process. It has been proposed that, upon the ligand interaction, Cav1 is activated through its phosphorylation regulation [27,28]. However, the regulation of Cav1 by phosphorylation is poorly understood in the field of the pathogen infection. In our studies, we have found several novel observations about Cav1 phosphorylation regulation. First, phosphorylation of Cav1 is enhanced in the presence of *C. neoformans*. The peak of phosphorylation is around 10 min at the fungal-host engagement (Figure 6), suggesting that Cav1 is involved during the *C. neoformans *infection. Secondly, the Cav1 phosphorylation is HA-dependent (Figure 7A), presumably, through the *C. neoformans *HA to HBMEC CD44 signaling pathway. Thirdly, phosphorylation apparently starts from the lipid rafts, as filipin treatment can reduce its phosphorylation level (Figure 7B); thus, its phosphorylation, most likely, triggers a dynamic internalization process there. Fourthly, a major finding of our study is that the Pi-Cav1 signal is observed as the thread-like structure inside the HBMEC, and this structure is colocalized with β-actin filaments, not with tubulin (Figure 8). It is known that Cav1 can associate with many proteins through its CSD domain, including actin-binding protein such as filamin [35,36]. In this scenario, activated Cav1 (Pi-Cav1) may be associated with β-actin through this binding domain and internalized into HBMEC. It is tempting to speculate that other signaling molecules may also be internalized via binding with Pi-Cav1 in a similar manner. Thus, Pi-Cav1 may carry certain signaling molecules with it during the internalization process. This speculation remains to be demonstrated.

## Conclusions

Our studies demonstrate that *C. neoformans *is able to manipulate the lipid-raft/caveolae endocytic pathway to facilitate its adhesion and internalization into HBMEC. Cav1 may function as a plasma membrane platform to localize caveolin interacting signaling molecules within caveolae membranes. Our results support a transcellular mechanism for *C. neoformans *invasion. We are currently investigating Cav1-associated signaling molecules and Cav1 regulation to unravel the profound invasion mechanism of *C. neoformans*.

## Abbreviations

BBB: blood-brain barrier; Cav-1: caveolin-1; CTxB: cholera toxin subunit B; HBMEC: human brain microvascular endothelial cells; HA: hyaluronic acid

## Competing interests

The authors declare that they have no competing interests.

## Authors' contributions

ML and CW performed immunofluorescence microscope. SHH carried out Western blots and statistical analyses. GMS studied the phosphorylation. AJ designed the experiments, and he is the principal investigator of this project. All authors read and approved the final manuscript.
